# Integrated Care in Aotearoa New Zealand 2008–2020

**DOI:** 10.5334/ijic.5679

**Published:** 2021-11-08

**Authors:** Jacqueline Cumming, Lesley Middleton, Pushkar Silwal, Tim Tenbensel

**Affiliations:** 1 Consultant Advisor, Te Hikuwai Rangahau Hauora – Health Services Research Centre, Faculty of Health, Te Herenga Waka – Victoria University of Wellington, Wellington, New Zealand; 2 Senior Lecturer, School of Health, Faculty of Health, Te Herenga Waka – Victoria University of Wellington, New Zealand; 3 Doctoral candidate, School of Population Health, Faculty of Medical and Health Sciences, University of Auckland, New Zealand; 4 Associate Professor, School of Population Health, Faculty of Medical and Health Sciences, University of Auckland, New Zealand

**Keywords:** integrated care, New Zealand, primary health care, equity

## Abstract

**Introduction:**

Ten years ago, progress towards integrated care in Aotearoa New Zealand was characterised as slow. Since then, there has been a patchwork of practices occurring under the broad umbrella of integrated care. These include: collective planning approaches (i.e., alliancing), agreed pathways of care, chronic care management initiatives, shared patient information systems, co-located centres and indigenous models of holistic care (e.g., Whānau Ora).

**Description:**

Although integrated care is often mentioned in national policy documents, implementation has been left to regional and local decision making, and very few initiatives have spread beyond their initial locations.

**Discussion:**

System incentives that preserve organisational “sovereignty” and path-dependent funding have slowed progress towards more integrated care in some areas. There is some evidence about specific initiatives and their impact, but it is difficult to discern significant trends and commonalities around the country.

**Conclusion:**

In the last ten years, the broad range of initiatives designed to achieve integrated care has absorbed regional and local attention and produced some evidence of progress, but the national picture of change is mixed.

## Introduction

Aotearoa/New Zealand (A/NZ) is generally regarded as having a high performing health care system, with universal coverage and generating good health outcomes at reasonable levels of expenditure. However, as with other countries, it faces challenges arising from an ageing population, increases in long term conditions, the development of new technologies, rising expectations, and significant inequities, which all need to be supported, while attempting to constrain expenditure and ensure good value-for-money.

In a 2011 paper on integrated care in A/NZ, Cumming (2011) [1] noted that a single, national, free and integrated health care system was the original ambition in the 1930s. This never eventuated; rather, what was implemented during the 1930s and 1940s were separately funded, owned, and organised health care arrangements across public health, primary, secondary, and community services. Thus:

- fully funded public health services were part of the national Department of Health, based in regional offices;
- partially subsidised primary care (PC) with co-payments was delivered through independent general practices owned by general practitioners (GPs), with pharmacies, laboratories and other diagnostic services also subsidised, and available through a GP referral;
- fully publicly funded hospital and some community care was provided by government-owned hospitals; and
- partially government funded community care also supported by public donations was delivered by a number of not-for-profit non-government organisations (NGOs) (e.g., well-childcare, ambulances).

Cumming (2011) [1] noted that, in the A/NZ case, ‘integrated care’ has been taken to mean the outcome of ‘integration’ (processes) from a service user perspective, involving more ‘co-ordinated’ care or a ‘seamless’ journey through the health system. Key forms of integration (a process for achieving integrated care) could focus on:

- ‘horizontal’ integration in PC;
- ‘vertical’ integration between PC and secondary care;
- ‘population health’ integration between health care and public health (e.g., screening and immunisation done through PC);
- ‘health and social services’ integration between health care and disability support and older people’s support services; and
- ‘intersectoral’ integration between health and other social development services (such as housing, employment, etc).

**Figure 1:**
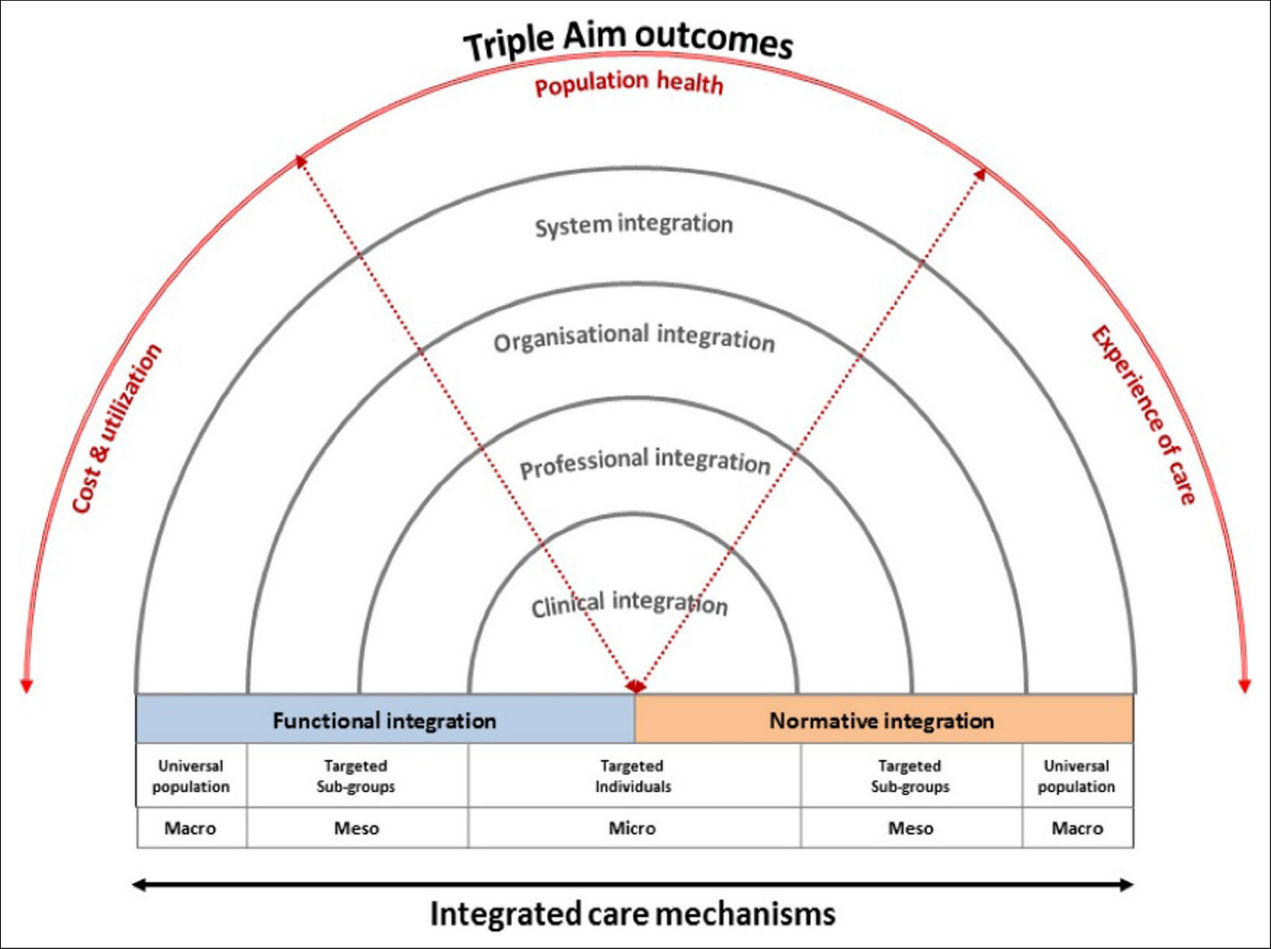
Conceptual framework of Integrated Care based on the integrative functions of PC [3].

Cumming (2011) [1] recognised that integrated services need to be supported by a “coherent set of methods and models on the funding, administrative, organisational, service delivery and clinical levels designed to create connectivity, alignment and collaboration within and between sectors” (adapted from [2]). This 2011 paper reviewed various A/NZ health system reforms to locate attempts at better integration at the micro (service delivery), meso (mid-system), and macro (system) levels, and according to planning and funding, service budgets, service and planning support, and service delivery functions. In terms of Valentijn’s Rainbow Model of Integrated Care (***Figure 1***) [3], the 2011 paper noted that most A/NZ reforms occurred via system and organisational reforms (especially mergers), while clinical (or service delivery) integration at the micro level has been far harder to achieve.

This article updates the material in Cumming (2011) [1]. This article first outlines, in Section 2, key features of the health and disability system and policy over the 2008–2020 period; we start in 2008 as that is when key integration policies that influenced policy up until 2020 began. During this 2008–2020 period, integrated care remained a core part of government policy, but with limited national drive and the onus on localities to design their own responses. In Section 3, we note how this has led to a resulting patchwork of local practices and we explore how a number of recent major initiatives have evolved in practice over time, along with a lack of spread of most initiatives. In Section 4, we explore findings from recent interviews that explain some of the patterns seen in Section 3. In Section 5, we draw overall conclusions about A/NZ’s recent forays in integrated care and look to the future following a recent review of the health system.

This article refers to Valentijn’s Rainbow Model of Integrated Care (***Figure 1***) throughout. The key means of integration considered particularly relevant in A/NZ include information sharing; service co-location; case management or care co-ordination; mutli-disciplinary team-work; shared planning and/or budgeting (including developing a shared vision, agreed care pathways, and agreed resource allocations); through to full organisational integration (via mergers). In this article, however, macro, meso and micro refer to levels relating to the formal structure of the health system.

One key area we do not focus on is that of Whānau Ora; policies and models of care influenced by Māori, the indigenous people of A/NZ, designed to take a more holistic view of health and wellbeing, and to integrate services intersectorally. Current Whānau Ora policies focus on improving the health and wellbeing of high needs whānau, especially Māori and Pacific families; both populations having significantly poorer health status and access to health care than other A/NZ populations. More detailed information on Whānau Ora is available in recent papers by Boulton [4] and Smith *et al*. [5]. Another area we do not focus on relates to recent policies to expand PC services through funding Health Improvement Practitioners and Health Coaches to support those with mild-to-moderate mental health concerns [6] and those needing assistance [7], for example, to improve their diets or increase their physical activity. These services are being integrated into existing PC services but are still relatively new.

## The Aotearoa/New Zealand Health and Disability System and Health and Disability Policy 2010–2020

**Figure 2:**
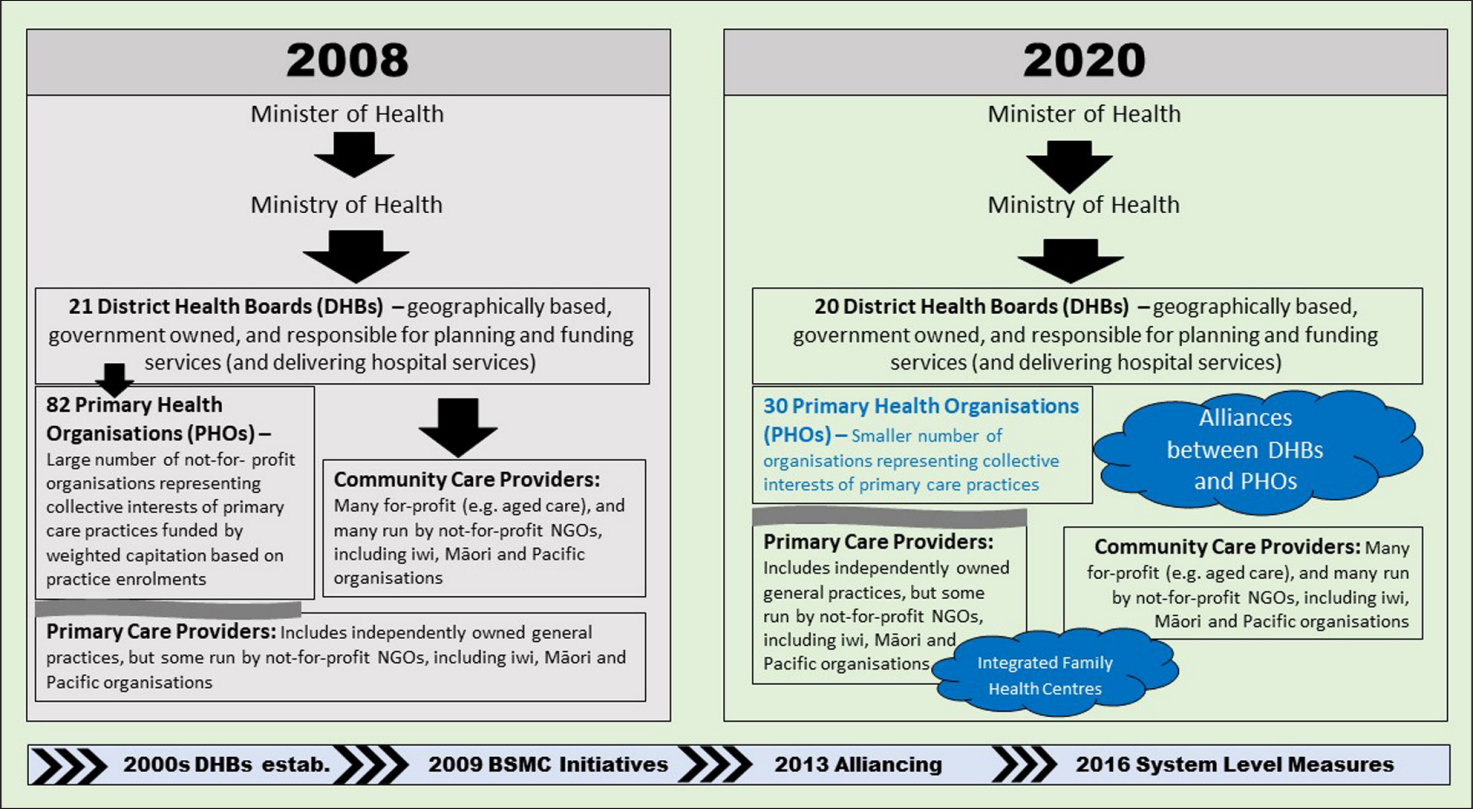
The Aotearoa New Zealand health system and integration in 2008 and 2020.

The A/NZ health system had its last significant reform in 2000, led by a newly elected Labour-Party-led Coalition government. The key structural arrangements put in place then remain largely in place in 2020. This is despite several changes of government, with a National-Party-led (conservative) Coalition government elected to power in late 2008 and governing until late 2017; a Labour-Party-led Coalition government formed in late 2017 governing until late 2020; and a sole Labour Party governing from late 2020 onwards. The key structural arrangements established prior to 2008 are laid out in ***Figure 2***. Overall, the A/NZ health system can be thought of as relatively well integrated at a system level, with predominant public funding and the vast majority of funding from a central source (the Ministry of Health or MoH); with single, geographically based planners and funders in the form of District Health Boards (DHBs); and in some districts, single meso-level Primary Health Organisations (PHOs) overseeing PC, enabling functional integration within PC (especially general practices), and supporting DHB and PHO collaborations.

Fragmentation occurs, however, where the MoH funds and contracts for some services nationally (e.g., well-childcare); and at the micro level where service users receive services from multiple professions and service delivery organisations, where services are not well co-ordinated.

Key policy directions (along the bottom), structural changes (compared horizontally across the two boxes) and integration activities (in clouds) between 2008 and 2020 are also set out in ***Figure 2***.

Policy in health care was largely driven between 2008 and 2017 by a 2007 National Party election manifesto, ‘*Better, Sooner, More Convenient Health Care*’ (BSMC) [8]. This included an emphasis on developing more person-centred care, to be delivered closer to home and to become more integrated, through greater collaboration within health and between health and social development services. The manifesto noted the need for more care to be delivered in general practices (e.g., diagnostics, minor surgery). There were also to be new Integrated Family Health Centres (IFHCs) which would co-locate and deliver services through multi-disciplinary teams of GPs, nurses, pharmacists, midwives, and allied health workers. Extended hours and diagnostics, day stay beds and observation beds would be included, shifting some hospital care into community settings. Some social care services (e.g., home and rest home care assessments and co-ordination, counselling, and social services) could also be available through IFHCs.

A 2009 Ministerial Review Group (MRG) similarly recommended: ‘new models of care which see the patient … at the centre of service delivery and which aim to promote a more seamless patient journey …, greater use of primary and community care, and the shifting of care “closer to home” [9, p.4]. The Group also recommended strengthening the management capability of, and reducing the costs of, PHOs, through PHO amalgamations. The BSMC and MRG policies began to be operationalised towards the end of 2009, with the supporting nine business cases (out of 70 applications), covering 60% of the population [10], to ‘deliver large scale changes’ in health care, to deliver care closer to home [11]. They included DHBs and PHOs working more closely together to plan and fund services, as well as, at the service delivery level, IFHCs, more nurse-led and nurse practitioner services, more multi-disciplinary teams, and greater co-operation between PC and hospitals. A key part of many applications was to reduce the number of PHOs, something which became policy in 2010 [12], leading to a significant reduction in the number of PHOs over time. The successful business cases did not receive new funding [13], but they were able to pool PHO funding streams into a ‘flexible funding pool’ [14]; such pools were later rolled out to the rest of the country. Over time, other business case applicants also started to implement their proposals. The nine BSMC business cases became known as ‘alliances’ [11].

In 2013, the government took the alliancing concept further and required each DHB to establish a District Alliance (hereafter, Alliance), at a minimum to include local PHOs, but also ideally other health care providers. Alliances were to involve both managers and clinicians, but as they were not legal entities, they could only make recommendations to each DHB Board, which then decides whether to implement and fund the recommended changes. Alliances have engaged in a number of activities, including developing service-level alliance teams to support service-level improvement initiatives and resource re-allocations [15].

Since 2016, Alliances have particularly worked on joint plans to improve System Level Measures (SLMs) within each DHB district. The System Level Measures Framework (SLMF) was introduced by the MoH with the purpose of stimulating local integrated care initiatives aimed at improving health outcomes and equity. Alliances were to develop SLM improvement plans, thereby facilitating inter-organisational collaboration to improve higher order performance measures [16]. There are six headline performance measures; four focused on integration between community, primary and secondary health care service providers (reductions in amenable mortality, total acute bed days, childhood ambulatory sensitive hospitalizations or ASHs, and improvements in patient experience of care); and two focused on integration between health services for babies and youth, delivered in community, primary and secondary care settings (babies in smoke-free homes, and youth health services). The SLMF policy intention is to build system integration from the bottom-up through organizational, professional and/or clinical (service) integration, and it has helped to stimulate some inter-organizational collaboration in health, and small-scale integration initiatives. However, after four years of implementation, there are mixed results in terms of its contributions towards achieving integrated care [17].

In 2016, the by then 15 years old 2001 NZ Health Strategy was ‘refreshed’. The ‘Future Directions’ refresh identified five key themes: that the system is: people-powered; delivering care closer to home; offering value and high-performance; delivering through a ‘one team’ approach; and ‘smart’. An ‘integrated and cohesive system’ working (both within health and between health and other agencies) ‘in the best interests of New Zealanders’ [p.15] are key themes throughout the document [18]. An accompanying ‘Roadmap of actions’ focused on a limited number of key areas, but, overall, was vague on how key aspects of integrated care would be achieved [19].

## A Patchwork of Local Practices

In line with Hughes and colleagues’ [20] contention that integrated care is best understood as a set of emergent practices shaped by local contexts, the nine initiatives initiated by the BSMC process covered a broad range of integrated care strategies. Each has had different trajectories as change ideas have evolved and merged with interests in new indigenous models of care, the sustainability of general practice, quality improvement and co-design approaches.

***Table 1*** displays details of five of the original nine businesses cases where there is a body of longer-term published material available. We have categorised the integration processes that have been adopted, using Valentijn’s Rainbow Model of Integrated Care as a guide [3], and, in reporting the results from each business case, we applied similar goals to that used by Goodwin *et al*. [21]. However, the lack of uniformity between initiatives underscores the challenge for evaluators over what counts as an integration intervention, and what populations have then been exposed to make judgements over the extent of change that has occurred.

**Table 1:** Local Integrated Care Initiatives.


	CANTERBURY	GREATER AUCKLAND	MID-CENTRAL	NATIONAL HAUORA COALITION	MIDLANDS

**Original Business Case Description**	**Canterbury Clinical Network** – a consortium of PC providers covering half a million people. The proposal focused on evolving general practice into IFHCs, developing the wider team of primary health care professionals, and improving cooperation between primary and secondary care	**Integrated Health Network** – a consortium of 274 general practice teams, 11 PHOs and 3 DHBs delivering PC to a million Aucklanders. The consortium was committed to working together to achieve better health outcomes, better patient experience and better use of money, establishing up to 12 IFHCs over time	**Four Mid-Central PHOs** (Ōtaki, Horowhenua, Manawatu and Tararua) proposed five Integrated Family Health Centres s, collaboration across health and social organisations, mainstream and Iwi providers, more clinical leadership, management of long term conditions, focus on care of the elderly, care of the young and care of those with mental health issues	**National Maori PHO Coalition** – involved 11 PHOs from around the North Island. The proposal aimed to devolve services and government-held resources to Māori communities. The Coalition aimed to develop a national network of whānau ora models of care including IFHCs, new care pathways, health and social service integration	**Midlands Network** – involved 11 providers from Taranaki, Waikato, Tairawhiti and Lakes districts which cover an enrolled regional population of around half a million people. The proposal identified consolidating $66 million worth of services that were purchased and managed by four of the Midlands region’s DHBs and their provider arms that could be devolved into the community. The plan also involved developing nine IFHCs

**Evolution**	Evolved into a wider Canterbury System change management and leadership programme under a ‘one system, one budget’ message developing new models of integrated working and new forms of contracting to support this. After several large earthquakes (2010/11), changes accelerated to relieve the immediate strain on the health service [33]	Evolved into a Localities initiatives in one DHB (Counties Manukau) which started with ambitious budget-holding proposals which were then abandoned due to lack of agreement between parties [39]. Attention then turned to building local relationships and implementing a long-term condition case management programme	Evolved into a diversity of local initiatives centred around different partnerships, and the building of Integrated Family Health Centres	Evolved into many initiatives adopting a whānau ora model of care including Mana Tū. As a result of a codesign process for Māori living with complex long-term conditions Mana Tū started with a focus on those with type 2 diabetes	Evolved into a Health Care Home Initiative modelled on the United States Medical Home model developed by Group Health Cooperative (tailored to the New Zealand context) [40]. Led initially by the ‘Network 4’ PHOs, the model has been picked up by others and governed by a Health Care Home Collaborative [41]

**Type of Integration**	Multi-level **system wide changes**– covering many categories of Valentijn’s rainbow model of care. For example: **Clinical Integration**: Older people case management support programme (CREST) **Professional Integration**: Multi-disciplinary guidelines including Health pathways and Acute Demand Management Programme supporting patients with a range of acute services in their own home **Functional Integration**: HealthOne (electronic Shared Care Record Viewer) **Normative Integration**: Investment in staff training and co-design workshops **Organisational Integration**: new forms of alliance contracting	Attempted multi-level **system-wide** changes but reverted to focus on **Clinical Integration** (case management and Multi-Disciplinary team meetings) for those at risk of hospital admissions] and **Functional Integration** (involving shared electronic medical record) Some **Normative Integration** through outreach clinics	Attempted complex multi-level system changes but reverted to making small scale local progress on; **Organisational Integration**: Building IFHCs **Professional Integration**: Integrated nursing pilots and partnerships with Whānau ora commissioning agencies.	Started as case management support within wider interest in indigenous models of care. Initial emphasis on: **Clinical Integration**: i.e. diabetes case management. From this starting base also weaves in: **Organisational Integration**: Shared governance of the service and creation of Network Hub; and **Professional Integration**: New skill mix in Kaimanaaki case workers **Functional Integration**: New digital information systems (Mohio) **Normative Integration**: Investment in early co-design workshops	Changes only at the level of the PC system involving: **Clinical Integration: E**xtended acute treatment options, multidisciplinary team meetings, and case findings and risk stratification **Professional Integration**: Increased capacity in general practice teams from Health Care Assistants and Clinical Pharmacists **Functional Integration**: Telephone assessment and triage, virtual consults, patient portals **Organisational Integration**: PC business change based on Lean processes

**RESULTS FROM EVALUATIONS [21]**					

**User and professional experiences**	Multiple evaluations and reviews highlighting the importance of leadership enablers including staff engagement, continuous quality improvement as well as technology able to drive a “one system one budget” approach [33,34,36]	Evaluation of what encouraged general practices to successfully implement more proactive care highlighted the importance of team approaches within practices which were prepared to change their organisational processes to support nurses to confidently take on new responsibilities for those with long-term conditions [40]	Evaluation found professionals ranked their perception of care coordination highly while patients rated their experience less highly. Success largely hinged on the enthusiasm of a small pool of frontline workers (champions) and their initial buy-into the idea of integrated care and a patient-centred approach(22]	Early assessments suggested user experiences were shaped by the way the Mana Tū programme was co-designed with whānau (patients and their families) to incorporate the experience of Māori experiencing long-term conditions. The philosophy of the Mana Tū programme is to support whānau to ‘mana tū’ (i.e., ‘to stand with authority’) [42]	Multiple evaluations highlighting the early energy and focus given to actions linked to improved business efficiency and sustainability of general practice. Staff generally rated the model higher than the traditional model of general practice [24], noting the changes heralded a call to action to move from a reactive PC model of care [24]

**Care outcomes**	Care outcomes limited to small studies. For example; integrated falls prevention strategies contributed to a reduction in harm from falls in the elderly population [34], and 78 per cent of a sample of clients who have been through the Community Rehabilitation programme (CREST) believed the service worked well with other health services in the home [36]	No specific data	No specific data	Early results found an average HbA1c decrease of 5mmol/mol for participants 3 months into the programme [43]. Cluster randomised controlled trial underway [42]	Population health targets (e.g. immunisations, smoking) were met. Patient portal use, and accessibility of appointments improved [44]

**Utilisation of services**	Findings related to bending the demand curve, “slowing – but not reversing – growth”. Results included: lower acute medical admission rates; lower acute readmission rates; shorter average length of stay; lower emergency department attendances [36]	An evaluation found no evidence of change that could be confidently attributed back to the Localities initiative from tracking secondary care demand across the Auckland region [40]	Analysis of routine date revealed that the desired 30% reduction in ASH rates were not realised. Data for ED presentations revealed a flat or slightly upwards trend [22]	No specific data	Early findings in one region have reported a significantly lower rate of ASHs (20% fewer) and a significantly lower rate of ED presentations (14% decrease) [24]. Another regional evaluation noted that while care was more accessible, timely, flexible and efficient, moves to more proactive care along with a focus on prevention and on patient centredness have not yet been strongly embedded [44]

**Financial performance/Cost effectiveness**	Compared with the rest of A/NZ, Canterbury has higher spending on community-based services; and lower spending on emergency hospital care	No specific data	No specific data	No specific data	The financial performance of Midlands-based Health Care Homes practices was reported to have been maintained or improved [24]. For Capital Coast-based Health Care Homes, an evaluation found initial funding to build capacity for change was useful but questions were raised as to whether funding now needs to be directed to support the capacity of communities to participate in a wider outreach stage [44]


Rather than a managed programme of change, the results highlight the staying power of initiatives which have adapted and evolved over time. One of the key lessons that emerges from this tracking is that multiple strategies for integrated care rise and fall within initiatives as change leaders seek to overcome organizational boundaries and professional scepticism. Detailed prescriptions of what should happen in the BSMC business cases may have initially secured an opening for workstreams directed towards integration. However, when short-term results did not reveal the expected results (e.g., reductions in ASH rates and ED presentations), efforts evolved towards implementing new models of service provision in response to the growing demands and pressures on PC [22,23]. The new Health Care Home model of care [24], for example, has now spread widely from its initial localities to other parts of A/NZ, although those involved would not necessarily describe it as an “integration” initiative, but as part of a wider reform programme focused on PC business practices.

Other adaptations of BSMC business cases included a recognition that integration initiatives may be a good fit for indigenous populations [25]. Initiatives such as Mana Tū, for example, responded to the need for culturally appropriate care to decrease health inequities. These initiatives drew on the ideas of indigenous scholars who advocate directing attention to the centrality of culture, identity and socio-economic factors for improvement in Māori health [26]. The resulting emphasis on relationship-based care involving psychosocial and cultural support has been seen most strongly in A/NZ in a new Whānau Ora service which incorporates cultural and relationship-based practices into the health system [4,5].

Although a number of evaluations of these local integrated care initiatives are available (as set out in ***Table 1***), these continue to be limited, particularly when it comes to assessing care outcomes, and to assessing whether changes are successfully bedded in over the medium- to long-term. Evaluations do, however, show the importance of leadership, including staff engagement, commitment, and team-work, as well as the need for adequate resourcing, including the freeing up of staff time, and training, in successfully bringing about change.

In addition to the BSMC business cases, Gurung *et al*. [27] identify a number of other PC focused A/NZ integrated care initiatives. A number of these were put in place in recent years, covering the 2008–2020 period discussed in this article. In addition to some of those models identified in ***Table 1***, and where there is information beyond the planning stage, they note an integrated care pilot relating to maternity care at three DHBs in place between 2014 and 2016; a shared care planning project based in Auckland and involving a large number of organisations; and Te Whiringa Ora, an integrated care initiative for those with long term conditions, in the Eastern Bay of Plenty. Their paper reinforces our finding of the existence of a patchwork of local initiatives, which do not appear to get beyond a pilot stage, and hence fail to spread. Evaluations of these initiatives similarly find communication between providers can be improved by such initiatives, but that poor relationships (and a lack of trust) between different providers, a lack of resourcing, and lack of training, are major barriers to achieving more integrated care.

The patchwork of initiatives seen in ***Table 1*** can be viewed in two ways. The first is that A/NZ has largely not succeeded in having a significant impact on established patterns of service delivery; without sustained spread of successful initiatives the experience of those receiving care will continue to be fragmented. Alternatively, the patchwork can be viewed as differently tailored responses to different community needs, where, in line with suggestions that integrated care is inseparable from context [20], local networks have made appropriate judgements on the need for particular types of integration and tailored initiatives for their different local populations.

## Insights from Recent Interviews on Integration

Insights into how judgements are being made about integration come from the preliminary findings of a research project investigating change in PC across A/NZ. These interviews have been undertaken as part of a wider programme of research on what works to support change in the delivery of PC across A/NZ. Those interviewed were responsible for making decisions on where PC resources are delivered and how, and the aim was to understand which changes have been able to be successfully implemented and sustained, and how governance, strategy and planning processes have supported such success.

The research used a realist research approach informed by Pawson and Tilley [28] adapted to assess a system-wide change rather than an evaluation of one specific change initiative. The realist approach aims to understand causation in a specific sense – that is, the mechanisms (resources and reasoning) that support change and that are triggered within specific contexts. The approach proceeds by developing initial programme theories and testing and refining these theories through iterative data collection and a search for underlying latent mechanisms.

A number of key themes were found to be relevant for programme theories surrounding PC reform in A/NZ – including PC policy in A/NZ reflecting countervailing structural powers of the state, the medical profession and (to a lesser, but increasing extent) business interests, and the importance of negotiation between power, politics and evidence [29]; the importance of change being driven from those working at the front-line, rather than being top-down; and the recognition that there would be policy conflict, i.e. contending frames amongst the different organisational health interests, with different values, knowledge, interests and narratives [30,31,32].

Fifty-five national-level, DHB and PHO leaders were interviewed during 2019 and 2020 for their views on where most and least progress had been made against 10 goals for PC reform over the last decade. Two of these goals related directly to integrated care: (1) better integration with secondary care and (2) improved collaboration with community-based services (health and non-health).

Interviewees were asked to indicate their level of agreement with four “if then” propositions of how change might occur to achieve each goal. For example, one proposition stated that “if DHBs and PHOs engage in collaborative approaches to planning and service re-configuration, then they are more likely to reduce avoidable hospital admissions in their district”. Responses were coded based on the extent to which interviewees agreed with these propositions. The analysis focused on distilling the modifying factors interviewees proposed to explain the circumstances that enabled or hindered progress.

Not surprisingly, given the diversity of experiences seen in A/NZ, interviewees were split on whether progress was being made. Some noted local progress, while others pointed to the enduring nature of known barriers such as: the lack of a shared electronic information system; a lack of trust between organisations; uncertainty over who takes responsibility for the socio-economic determinants of health; and a lack of leadership.

In some local areas, Alliances, networks, specific initiatives, and clinical engagement had clearly created a sense of momentum. This is most evident in the evaluations of the Canterbury experience [33,34,35,36], which has been credited with moderating demand for hospital care. The processes involved have included leadership around a “one system one budget message”, investment in staff skills, new forms of contracting, new referral pathways, shared technologies, and case management programmes. But while the Canterbury Clinical Network BSMC initiative has matured into a local system-wide integrated model of care, others found freeing up resources to do more in PC, “easy to talk about but harder to do”, and it was not uncommon for other integrated care initiatives to be recalled as “projects” involving many meetings that only fleetingly influenced day-to-day operations. Frustration was expressed that despite energy and effort put into projects designed to better integrate primary and secondary care, most had yet to become business-as-usual.

When interviewees reflected on how much progress had been made towards improved collaboration with community-based services, as opposed to secondary services, a number noted that faster progress is being made by those PHOs who had a starting organisational philosophy that this is “my responsibility”. In response to recognising the importance of tackling the socio economic determinants of health if significant improvements in health and reductions in inequities were to be made, these PHOs and their member practices were more likely to engage in projects requiring practice staff to think about their role as navigators of services rather than solely providers of medical care.

Interviewees gave examples of the type of activities PHOs could do which could make collaborations “easier” for general practices. These ranged from simple mapping of potential services to much more sophisticated referral pathways and decision tools enabling general practices to fit collaborative activity into their busy workflow. Others gave a stronger emphasis on the relational nature of collaboration with an example of how one PHO forged personal relationships between one mental health NGO and one practice in an environment where there were many competing mental health NGOs. The result was that:

*Through having that personal relationship people were much more likely to refer into those services. Now the services weren’t always the right service, but the NGOs had a better understanding about what each other did differently to move the people around than trying to teach all of the GPs what all of the NGOs do. (PC leader)*

These relational efforts are not widespread. As other A/NZ research has found, the degree to which general practices have moved outside medical concerns and taken on a broader role in horizontal and intersectoral integration has been limited [37]. Given that many PHOs developed and remained largely as GP-owned and -focused organisations, PHOs can only move as fast as their general practices are comfortable with in terms of embracing responsibility for the wider socio-economic determinants of health. The wider setting for PC in A/NZ also plays a role as reliance on co-payments continues to incentivise delivering numbers of consultations rather than deeper, proactive care [23].

A distinguishing feature of the wider commentary from those interviewed in the PC reform research project was the recognition of a lack of progress and future importance of overcoming health inequities between Māori and non-Māori. The unresolved question is, will more local integrated care initiatives lead to a reduction in inequities as part of a shift to person-centered integrated care, or divert effort from the call to hold the many layers in the health system accountable for improved outcomes, addressing racism, and strengthening the participation of Māori in policy-making [38]?

*Yeah so you get to the point well why do you spend all that energy and effort? You know it’s important but is there another way of cracking the egg? Can we get the outcomes we desire by working with our communities, working with Māori and doing those things rather than having this holy grail of integration? (PC leader)*

## Discussion and Conclusions

A/NZ governments have continued over recent years to promote integration as a key goal of the health system. However, the most obvious changes have continued to occur in relation to system and organisational integration, in the form of functional integration, at the macro and meso levels, where the government has more control (e.g., using funding levers to encourage PHOs to amalgamate; requiring DHBs to establish Alliances; requiring Alliances to develop SLM improvement plans). Having fewer PHOs as meso level organisations working with their member PC providers could make it easier for DHBs to develop more collaborative working arrangements with PC, as might the development of Alliances, but research has shown that DHBs, PHOs and Alliances have varied widely in their interest and capacity to pursue integration, particularly when it comes to integration with community and social services.

The lack of an overall plan for achieving integrated care especially in terms of service delivery reflects the light touch policy environment that has characterised PC reform in A/NZ [23]. In this environment, integrated care policy has generally been ‘bottom up’ with no central blueprint. Rather, DHBs and PHOs have been encouraged to come together in different configurations to test out different types of integrated care approaches. The overall result is a patchwork of local initiatives, built through the BSMC applications and business cases in the early 2010s, and through each Alliance’s response to the SLMF in the later 2010s.

Health systems are complex adaptive systems that rely on local sensemaking to devise solutions and attempts to prescribe change can be counter-productive [45]. There being no detailed national direction has had the advantage of allowing for local bottom-up, context-specific initiatives, and these have typically linked integration with ideas involving enhanced PC services, long-term condition management, quality improvement and whānau-centred models of care. On the other hand, it is not entirely clear how much these initiatives stimulate others to take up new ideas, and in most cases key initiatives do not appear to spread throughout A/NZ. This may in part be because of the continued differences in funding arrangements across the system, including in PC, which continues to incentivise acute presentations.

The energy going into integration appears to have waned in the latter part of the 2010s, potentially attributable to an increasingly tight financial environment, which does not support continued experimentation in relation to integration. Even the Canterbury success may now be at risk, due to its financial position and significant changes of leadership, including the appointment of a Crown observer on the DHB Board [46].

There is an increasing realisation internationally that integration is a highly complex, multi-faceted concept, with multiple goals at many levels making overall evaluation of progress difficult. The variant approaches taken in A/NZ demonstrate this clearly. A/NZ therefore cannot continue to talk vaguely about better integration; at all levels, there is a need for a much clearer assessment of the problems and types of integration that system reform and new models of care are trying to solve, if progress is to be made. As noted by Cumming (2011) [1], there remains the need for more evaluation and spread of successful initiatives, where similar contexts exist across the country.

The A/NZ experience demonstrates that, at a national level, much more work needed to be done to be clearer about the specific problems raised by the delivery of fragmented services, using a range of examples across the myriad services delivered across the A/NZ health and disability system. A clearer framework was needed in terms of how services might become better integrated, again using a range of examples, and setting solutions within the context of structural and organisational arrangements of the A/NZ system. Sustained leadership, adequate resourcing (including of people’s time), and evolutionary strategies guided by more in-depth and longer term monitoring and evaluation might also have better supported the achievement of the goal of delivering more integrated services over time in A/NZ. There also needed to be better recognition of some of the major constraints that might limit better integration – in particular, the role that user charges continue to play and that provide significant barriers to access to PC services, the need for PC services to expand over time to provide a supportive context for a stronger role in integrating or co-ordinating services, and a recognition that PC services continue to fail to deliver appropriate services to higher needs groups, in particular Māori and Pacific populations.

A recent Health and Disability System Review has seen two reports released (Interim and Final) which provide a diagnosis of the key issues facing the A/NZ health system, and recommendations for how to address them [47,48]. As a term, ‘integration’ does not feature prominently in either report, even though fragmentation is frequently identified as a problem concerning the planning and delivery of health and disability services. However, many of the suggested solutions can be understood in terms of Valentijn’s model of integration, albeit particularly again at the system and organisational levels, and in terms of functional integration. Integration between primary and secondary care is particularly emphasised in the reports, including through better information flows.

The government recently released its response to the Health and Disability System Review [49]. The government is firstly planning to streamline the role of the Ministry of Health, strengthening its public health role, and removing its service purchasing/commissioning role. Secondly, it is planning to fully integrate all DHBs into a single, national health service, through a new body to be called Health New Zealand. Health New Zealand will run all hospital and hospital-related community services, including regional public health units, and will purchase/commission a wide range of PC and community services. It will have four regional offices and a range of district offices. Thirdly, there will be a greater emphasis on working with local communities to better meet health needs through Health New Zealand establishing a range of locality networks, the details of which have yet to be determined. These networks are expected to go beyond traditional GP services, and to achieve more co-ordinated care. PHOs will not have a formal place in the structure as they do now. Finally, a new Māori Health Authority is also to be established, working with the Ministry of Health and Health New Zealand to improve Māori health through planning, policy and purchasing/commissioning, and with its own funding to commission services. The new structures are due to be in place by 1 July 2022.

These reforms continue A/NZ’s focus on reorganising the health sector via the organisations that the government owns and controls (i.e., Ministry of Health, DHBs and Health New Zealand, and the new Māori Health Authority). That the localities aspects of the reforms are the least developed is no surprise when the government has less control over what occurs at the local level, with services owned and controlled by a wide range of privately owned organisations.

Varying hospital service delivery arrangements across DHBs and the extremely slow developments in PC service delivery, including the lack of attention being paid to, and lack of success in achieving, more co-ordinated care at a PC level, sit behind the Minister of Health’s decision to dismantle the current system rather than to build on existing arrangements. The reforms on the one hand streamline national planning and purchasing/commissioning processes and integrate those processes with hospital and hospital-related service delivery, through the establishment of Health New Zealand. On the other hand, a degree of fragmentation arises through the establishment of a Māori Health Authority, but this is urgently needed to better support more equitable service delivery and outcomes for Māori. Primary care and community services purchasing/commissioning continues to be separate from actual service delivery, and such services continue to be delivered by a raft of privately owned health care providers, although more co-ordinated care through localities is a key goal of the reforms. However, the details on localities are yet to be determined.

If major reforms had not been planned, we surmised that A/NZ would continue to see small scale changes being pursued at local levels, as part of person-centred improvements in care emerging from increasingly co-designed processes rather than changes being driven by national blueprints. Health New Zealand may, however, bring a stronger focus to achieving more integrated care in the future. However, the time needed to establish new national organisations and getting them operational over the next few years is likely to mean that A/NZ will not see any further developments in integrated service delivery for some time yet. That said, recognising the time needed for the new organisations to bed in, the Cabinet paper on the changes stressed the importance of seeing a programme of early implementation activities demonstrating how the reformed system will work. It will be interesting to see whether this early programme becomes the route to spread any of the successful pockets of activity we have outlined.
